# A Novel Detection Refinement Technique for Accurate Identification of *Nephrops norvegicus* Burrows in Underwater Imagery

**DOI:** 10.3390/s22124441

**Published:** 2022-06-12

**Authors:** Atif Naseer, Enrique Nava Baro, Sultan Daud Khan, Yolanda Vila

**Affiliations:** 1 ETSI Telecomunicación, Universidad de Málaga, 29071 Malaga, Spain; en@uma.es; 2 Science and Technology Unit, Umm al Qura University, Makkah 21955, Saudi Arabia; 3 Department of Computer Science, National University of Technology, Islamabad 44000, Pakistan; sultandaud@nutech.edu.pk; 4 Centro Oceanográfico de Cádiz (IEO-CSIC), Instituto Español de Oceanografía, 11006 Cádiz, Spain; yolanda.vila@ieo.csic.es

**Keywords:** *Nephrops norvegicus*, deep learning, detection refinements, spatial–temporal analysis

## Abstract

With the evolution of the convolutional neural network (CNN), object detection in the underwater environment has gained a lot of attention. However, due to the complex nature of the underwater environment, generic CNN-based object detectors still face challenges in underwater object detection. These challenges include image blurring, texture distortion, color shift, and scale variation, which result in low precision and recall rates. To tackle this challenge, we propose a detection refinement algorithm based on spatial–temporal analysis to improve the performance of generic detectors by suppressing the false positives and recovering the missed detections in underwater videos. In the proposed work, we use state-of-the-art deep neural networks such as Inception, ResNet50, and ResNet101 to automatically classify and detect the Norway lobster *Nephrops norvegicus* burrows from underwater videos. *Nephrops* is one of the most important commercial species in Northeast Atlantic waters, and it lives in burrow systems that it builds itself on muddy bottoms. To evaluate the performance of proposed framework, we collected the data from the Gulf of Cadiz. From experiment results, we demonstrate that the proposed framework effectively suppresses false positives and recovers missed detections obtained from generic detectors. The mean average precision (mAP) gained a 10% increase with the proposed refinement technique.

## 1. Introduction

Research in underwater image analysis has gained popularity in many applications of marine sciences. There are various research directions in underwater image analysis, for instance, underwater species classification and detections [1], seafloor image recognition [2], coral reef classification [3], and flora and fauna recognition [4]. Underwater image analysis requires a set of image processing tasks including underwater object detection, classification, visual content recognition, and image annotation of large-scale marine species [5]. Certain challenges such as turbidity, color variations, and illumination changes make underwater environments very difficult for the models to detect and classify the objects automatically.

**Figure 1:**
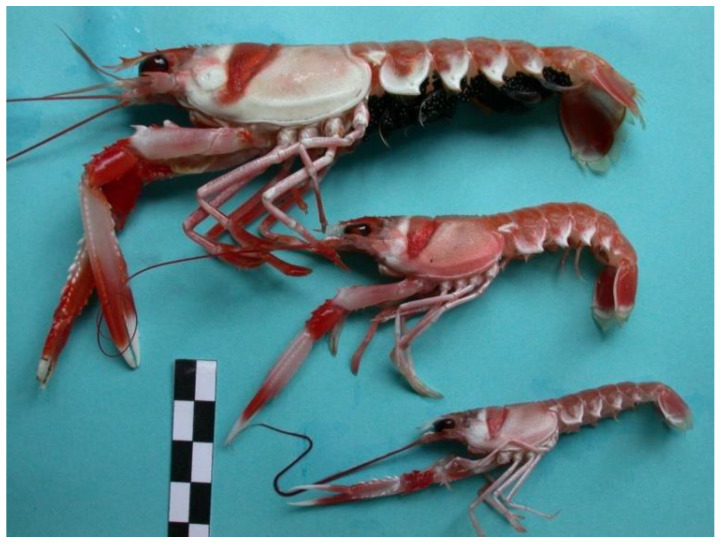
Some individuals of *Nephrops norvegicus*.

**Figure 2:**
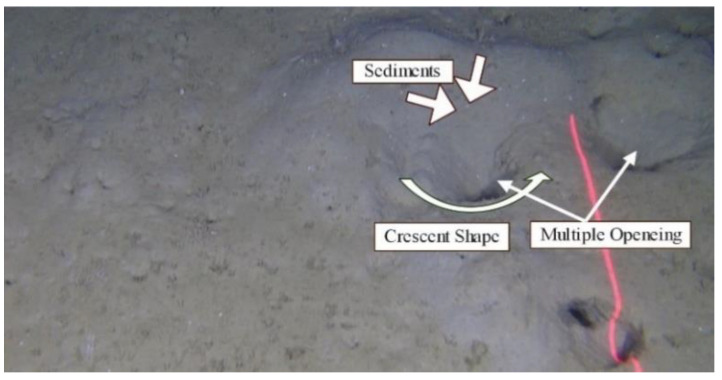
*Nephrops* burrow system.

There are thousands of species in the ocean all over the world. One of the most important commercial species in Europe is the Norway lobster *Nephrops norvegicus*. Figure 1 shows the *Nephrops norvegicus* species (hereafter referred to as *Nephrops*). This species is distributed from 10 m to 800 m of depth in the Atlantic NE waters and the Mediterranean Sea [6], where sediment is suitable for them to construct their burrows. This species excavates into and inhabits burrow systems mainly in muddy seabed sediments, with more than 40 percent silt and clay [7]. These burrows systems have a single or multiple openings or holes with characteristic features that make them different to burrows built for other burrowing species [8,9]. At least one opening has a crescent moon shape and a shallowly descending tunnel. It is often proof of expelled sediment forming a wide delta-like tunnel opening, and signals such as scratches and tracks are frequently observed. If a burrow system consists of more than one entrance, then the center of all the openings has a raised gain. It is assumed that each burrow system is occupied by a unique individual. Figure 2 shows the features of the *Nephrops* burrows system.

*Nephrops* spend most of their time inside the burrows, and their emergence behavior is influenced by several factors: time of year, light intensity, or tidal strength [10]. For this reason, abundance indices obtained from the commercial catch or the traditional bottom trawl surveys are thought to be poorly representative of the *Nephrops* population and they are not considered appropriate [11,12].

The abundance of *Nephrops* populations is currently monitored by underwater television (UWTV) surveys on many European grounds. The methodology used in UWTV surveys was developed in Scotland in the 1990s and is based on the identification and quantification of the burrows systems over the known area of *Nephrops* distribution [13]. *Nephrops* abundance from UWTV surveys is the basis of assessment and advice for managing these stocks [14].

Videos are recorded using a camera system mounted in a sledge with angle with respect to the bottom ranging between 37–60° depending to the country [15]. They are reviewed manually by trained experts and quantified following the protocol established by ICES [8,16].

With the recent advancement in artificial intelligence and computer vision technology, many researchers employ AI-based tools to analyze marine species. Some people use feature extraction mechanisms to count and identify the species while others use some advanced techniques [17] such as neural networks. Convolutional neural networks (CNN) bring a revolution in object detection. Deep convolutional neural networks gain tremendous success in the tasks of object detection [18,19], classification [20,21], and segmentation [22,23]. These networks are data-driven and require a huge amount of labeled data for training.

In our previous work [24], we developed a deep learning model based on state-of-the-art Faster RCNN [19] models Inceptionv2 [25] and MobileNetv2 [26] for the detection of *Nephrops* openings. Those models were trained on Gulf of Cadiz and Irish datasets. These models achieved good results in detecting the burrows from the image test data. However, when these trained models were tested on a video from Gulf of Cadiz, the accuracy of the detectors degraded. We figured out many false positive (FP) and missed true positive (TP) detections that adversely affect the accuracy of these models.

**Figure 3:**
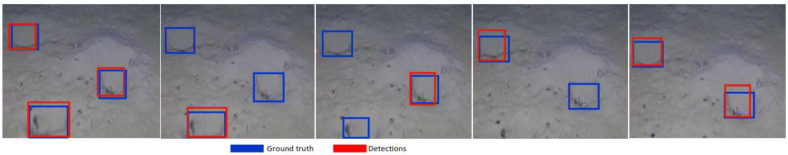
Ground truth (blue color, bounding boxes). The result of detector (Inception) (red color, bounding boxes). Due to camera angle variation and burrows appearance, the detector missed detections in consecutive frames.

In this work, we proposed a detection refinement mechanism based on spatial–temporal information to enhance the detection of missed true positive and suppress the false positive detections. The work presented in [27] used the temporal information to track the faces and suppresses the false positive detections. Their approach used low-level tracking to detect the faces in real images. Furthermore, their approach does not recover the missed detections. In our case, the low-level tracking cannot be applied as we are using underwater videos and the objects we are detecting are not real species but the burrows on the ground, where the characteristics are very different than the natural image. The previous work integrates the temporal information to track the faces and suppress the false positives. In our approach we are using the spatial and temporal information to suppress the false positives and recover the missed detections. Our work is divided into two parts. At first, we trained the model using state-of-the-art Faster RCNN [19] models Inceptionv2 [25], ResNet50 [28], and ResNet101 [29] for the detection of *Nephrops* burrows. We built the dataset for training and testing the models. In the second part of our work, we presented a spatial–temporal-based detection refinement algorithm. We detected the burrows in each frame in a video sequence and then obtained the spatial and temporal information across the multiple frames to refine the *Nephrops* burrows detections. The spatial–temporal mechanism helped in suppressing the FP burrows and allowed us to find the missed TP detection that led us to achieve a better accuracy as well as tracking and counting burrows in a video sequence. Figure 3 shows the result of the detector that we trained using the Inception model. The bounding boxes in blue color show the ground truth, while the red color bounding boxes show the detections from the Inception model. Due to variation in camera direction and appearance of burrows, the detector accumulates FPs and missed detection in some frames. The figure clearly shows the missed detection in the intermediate frames.

To address these challenges, we proposed a detection refinement approach based on spatial–temporal analysis that enhances the mAP of a generic detector. Our proposed detection refinement mechanism identified these missed detections, recovered them, and suppressed the false positives. Generally, our approach has the following contributions:

i. We propose the spatial–temporal filtering (STF) model that extracts the spatial and temporal information of all the detections of the consecutive frames of an input video by suppressing the false positives and recovering the missed detections. The proposed method will improve the performance of the generic detectors (such as Inception and ResNet, in our case).
ii. We evaluate the performance of the proposed framework on our proposed novel dataset. From the experiment results, we demonstrate the effectiveness of the proposed approach.

The rest of the paper is organized as follows: the related work is presented in Section 2. The Materials and Methods section given in Section 3 presents the data collection method and proposed methodology to refine the detections. The achieved results with the proposed methodology are discussed in Section 4. Finally, Section 5 concludes the article.

## 2. Related Work

Object detection and classification is a challenging computer vision problem. Researchers have developed many methods for object detection and classification tasks. The existing object detection approaches use handcrafted feature-based models [30,31,32,33] and deep features models [34]. The hand-crafted features models use basic features such as shape [35], texture [36,37,38], and edges [35,38] to train the classifier. On the other hand, convolutional neural networks automatically learn hierarchical features from the training set. Deep learning replaces the handcrafted features and introduces some efficient algorithms for object detection and classification. Over the last few years, deep learning models have enjoyed tremendous success in various object detection and classification tasks. Due to this reason, deep learning models are also employed in the detection and classification of underwater species. Although the underwater environment is hard and challenging compared to the ground, the deep learning algorithms perform much better compared to the conventional and handcrafted features. State-of-the-art deep learning-based object detectors include region-based convolution network (R-CNN) [39], Fast R-CNN [40], and Faster R-CNN [19]. R-CNN uses deep ConvNet to classify the object proposals. R-CNN algorithm is computationally expensive as it uses a selective search [41] strategy to generate a large number of object proposals followed by the object proposal classification step. On the other hand, Fast R-CNN is the improvement of R-CNN, where a faster training process is achieved compared to R-CNN. Fast R-CNN uses multitasking in updating all the network layers and handling the loss which improves the speed and accuracy of the network. Compared to both methods, Faster R-CNN introduces region proposal network (RPN) as it combines the RPN with Fast R-CNN into a single network.

Li et al. [42] developed a deep learning model for the detection of marine objects. The model detects and recognizes fishes using deep convolutional network. They applied the Fast R-CNN algorithm to classify the twelve different classes of underwater fishes. They also introduced a dataset of 24,272 images of all these classes. They achieved more than 90% of accuracy in detection. Similarly, Villon et al. [43] applied the deep learning algorithms to the Fish4Knowledge dataset project to detect and classify the fishes. Rathi et al. [44] combined Faster R-CNN with three classification networks (ZF Net, CNN-M, and VGG16) to detect 50 fish and crustacean species from Queensland beaches and estuaries. The regional proposal method consists of a regional proposal network coupled with a classifier network. Xu et al. [45] applied the YOLO deep learning model to recognize the fishes in underwater videos. They used three different types of datasets that were recorded at real-world waterpower sites. They achieved an mAP up to 53.92%. Mandal et al. [46] presented a Faster R-CNN approach to identify the fishes and their different species using deep neural networks. Gundam et al. [47] also proposed a fish classification technique based on the Kalman filter that used partial automation of fish classification from underwater videos. Jalal et al. [1] proposed a hybrid approach that combines the YOLO-based object detection with optical flow and Gaussian matrix models to detect and classify the fishes from underwater videos. A similar method based on YOLO to detect and classify the fishes was proposed by Sung et al. [48]. They used 892 images and achieved the fish classification accuracy up to 93%. Jager et al. [49] proposed a deep CNN approach based on AlexNet architecture for the classification of fish species. They used the dataset of LifeCLEF 2015. Zhuang et al. [50] proposed a deep learning model based on SSD detector to automatically identify the fishes and their species. In their approach they used ResNet-10 as a classifier for species identification. Zhao et al. [51] proposed an automatic detection and classification method for fish and underwater species. The proposed method, called “Composed FishNet”, is based on the composite backbone and a path aggregation network. The composite backbone method is the improvement of ResNet. The enhanced path aggregation network is designed to improve the semantic information caused by unsampling. The results show that they achieved an average precision (AP) of 75.2%. Labao et al. [52] proposed a multilevel object detection network that used R-CNN as network framework. Their proposed network contained two region proposal networks and seven CNNs connected by long short-term memory (LSTM). The proposed network showed an improvement in the performance over the simple one-stage detection networks. Salman et al. [53] proposed an R-CNN-based two-stage automatic fish detection and location method. They used the fish motion information and combined it with the background and optical flow information to generate the candidate region of the fish. Their proposed model requires a fixed size input image and the candidate region extraction needs a substantial disk space as well.

Deep learning models also have been employed to detect marine objects other than fishes, such as planktons and corals. These two are also major components of the underwater marine ecosystem. Plankton are the basics of aquatic food. Dieleman et al. [54] used a deep neural network to classify the plankton. They introduced the inception module for image information extraction. Lee et al. [55] also proposed a deep neural network for plankton classification on a large dataset. Their convolutional neural network used three convolutional layers and two fully connected layers. The problem with the coral classification is its color, size, texture, and shape. Shiela et al. [56] introduced a local binary pattern for texture and color coordination. For classification purposes, they used the neural network with three backpropagation layers. Elawady et al. [57] used supervised CNN for the classification of corals. Table A1 in Appendix B summarizes the key findings of the papers discussed in this section.

## 3. Materials and Methods

**Figure 4:**
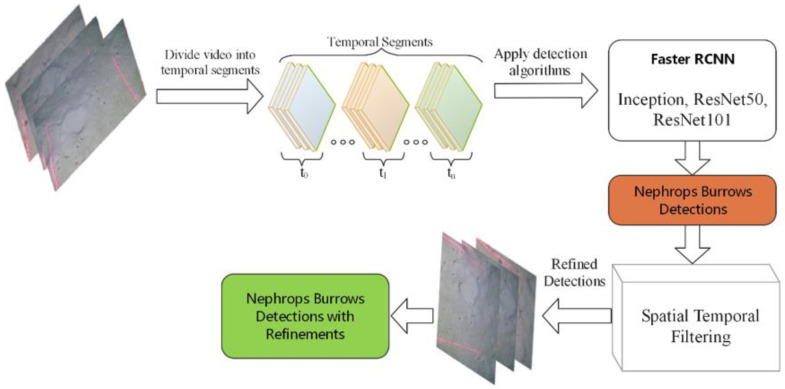
Detection refinement framework based on spatial–temporal filtering.

In this section, we discuss the proposed methodology of improving the detections of *Nephrops* burrows. Figure 4 shows the pipeline of proposed framework. This section also presents the equipment and method used in the data collection in detail. Generally, the proposed framework has two sequential stages. The first stage is object detection, while detection refinement is performed during the second stage. During the first stage, we use state-of-the-art generic detectors, for example, Faster RCNN, Inception, ResNet50, and ResNet101, to detect the *Nephrops* burrows. For this purpose, we first divide the input video sequence into temporal segments, with each segment consisting of *N* number of frames. We then apply state-of-the-art detectors to each temporal segment to detect *Nephrops* burrows. The obtained results are passed to the refinement module that will employ spatial–temporal filtering (STF) to recover the missed detections from the frames and suppress the false positive detections. This process improves the mean average precision (mAP) of the results obtained from the detectors.

### 3.1. Nephrops Burrows Detections

**Figure 5:**
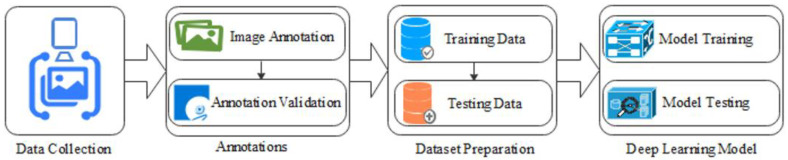
*Nephrops* burrows detection framework.

To detect and classify the *Nephrops* burrows, state-of-the-art Faster R-CNN deep learning algorithms, Inceptionv2 [25], ResNet50 [28], and ResNet101 [29], were used to train the models. Figure 5 shows the pipeline of the proposed detection framework.

#### 3.1.1. Data Collection

**Figure 6:**
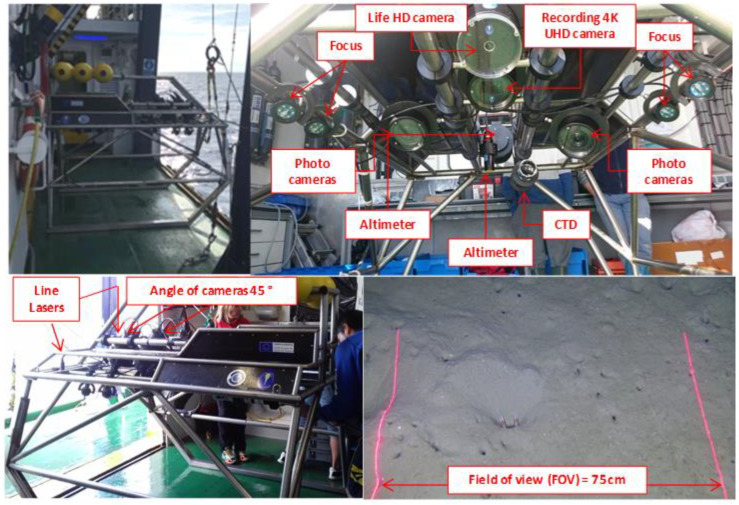
Sledge and equipment use in 2018 UWTV survey at the Gulf of Cadiz.

High-resolution footage was collected using a sledge during the 2018 Underwater TV (UWTV) survey at the Gulf of Cadiz by marine scientists who belong to IEO (Instituto Español de Oceanografía), a Spanish research institution devoted to promoting ocean research and knowledge, including government assessment for sustainable fisheries. A sledge is a stainless-steel underwater vehicle equipped with multiple cameras, sensors, lasers, and lights to record the footage. Figure 6 shows the setup of the instruments mounted in the sledge and a sample image, and a complete description is presented in Table 1.

Sampling on 70 stations were conducted in the 2018 UWTV survey. A station is a geostatistical location where the *Nephrops* burrow density is estimated to obtain the *Nephrops* abundance index over the known survey area using geostatistical analysis. At each station, the sledge was deployed and towed with constant speed between 0.6–0.7 knots to obtain the best possible conditions for counting *Nephrops* burrows. Once the sledge is stable on the seabed, a video footage of 10–12 min at 25 frames per seconds is recorded, which corresponds to 200 m swept, approximately. Vessel position (dGPS) and position of sledge, using a HiPAP transponder, are recorded every 1 to 2 s. The distance over ground (DOG) is estimated from the position of sledge in all stations, and the field of view of the video footage is 75 cm (FOV), which was confirmed using two line lasers. Out of all these 70 stations, we selected seven based on the better lighting conditions, high contrast, and high density of *Nephrops* burrows, as well as the better visibility of burrows. The recorded footages were saved into hard disks for further analysis on *Nephrops* density.

#### 3.1.2. Image Annotation

The obtained frames were annotated using Microsoft VOTT [58] tool. We adopted the mechanism to annotate the burrows manually in the Microsoft VOTT image annotation tool and saved the annotations in Pascal VOC format. The saved XML annotation file contains image name, class name (*Nephrops*), and bounding box details of each object of interest in the image. The annotated frames led to formulating the ground truths (GT) for model training. To create the datasets for training and testing, from the set of annotated frames (more than 100,000), we selected those which contained *Nephrops* burrows, using the criteria of using only one frame per individual object, selected to increase the diversity of its appearance, which the aim of creating a small dataset which contained most of the typical cases of *Nephrops* burrows.

#### 3.1.3. Annotation Validation

The *Nephrops* burrows annotation is a tedious job, and it requires a lot of experience to annotate a burrow, because different species build burrows with similar appearance on the bottom of the sea. Once all the burrows are annotated, it is very important to validate each one of them with the advice of marine experts from IEO institution, Gulf of Cadiz. Only the validated annotations were used in the model training.

#### 3.1.4. Prepare Dataset

After validating all the annotations, the dataset was divided in two independent groups, the first one for training and the second one for testing purposes. Details are given in Table 2.

#### 3.1.5. Model Training

We utilized transfer learning [59] to fine-tune the models in TensorFlow [60]. Inceptionv2 [25] is one of the architectures that have a high degree of accuracy, which helps to reduce the complexity of CNN. Inceptionv2 has 3 × 3 convolutions layers, which increases the performance of the network with respect to computational speed and processing.

ResNet50 [28] is a variant of the model ResNet. The ResNet50 has 48 convolutional layers, one max pool, and one average pool layer so it is a 50-layers-deep convolutional network. Out of these 50 layers, one layer is used in the first convolution with a kernel size of 7 × 7 64 kernels with stride 2 and a max pool of size 3 × 3 with stride 2, nine layers are used in the second convolution with a kernel size of 1 × 1, 64 kernels and 3 × 3, 128 kernels. In the next step, 12 layers are used with 1 × 1, 128; after that, a kernel of 3 × 3, 128, and, at last, a kernel of 1 × 1, 512. The fourth convolution uses 18 layers with kernel of 1 × 1, 256 and two more kernels with 3 × 3, 256 and 1 × 1, 1024. The fifth convolution uses nine layers with 1 × 1, 512 kernel with two more of 3 × 3, 512 and 1 × 1, 2048. Finally, the last layer is used for avg pool and a softmax function. ResNet50 is a widely used ResNet model.

The ResNet101 [29] is a dense convolutional neural network that is 101 layers deep. The first convolution has a kernel size of 7 × 7 64 kernels with stride 2 and a max pool of size 3 × 3 with stride 2. Nine layers are used in the second convolution with a kernel size of 1 × 1 64 kernels and 3 × 3 128 kernels. In the next step 12 layers are used with 1 × 1, 128; after that, a kernel of 3 × 3, 128, and, at last, a kernel of 1 × 1, 512. The fourth convolution uses 69 layers with kernel of 1 × 1, 256 and two more kernels with 3 × 3, 256 and 1 × 1, 1024. The fifth convolution uses 9 layers with 1 × 1, 512 kernel with two more of 3 × 3, 512 and 1 × 1, 2048. Finally, the last layer is used for avg pool and a softmax function. The ResNet50 and ResNet101 have better accuracy when compared to the other models for our problem.

#### 3.1.6. Testing

To test our algorithm, we selected another station from the Gulf of Cadiz whose frames were not used in the training dataset. The test video, which is five minutes long and contains 7500 frames, was divided into temporal segments and then passed to our trained models to obtain the *Nephrops* burrows detections.

### 3.2. Detection Refinements

After the detections of *Nephrops* burrows, we performed a post analysis of the obtained results. After a critical analysis of the results, we found that the detectors encounter many FP and missed many TP, which degrades accuracy. To recover missed detections and suppress FP, we propose a detection refinement algorithm that exploits the spatial–temporal information among consecutive frames of the given temporal segment. The Inception, ResNet50, and ResNet101 models are tested on a video of five minutes in length. The proposed detection refinement algorithm takes *V*, λ, and W as inputs, where *V* is the video, λ, is a threshold value for displacement vector, the threshold value is the value of IoU (intersection over union) that is compared later with the IoU of detected *Nephrops* burrow, and W is a size of temporal window which determines the number of frames in the temporal window. These models provide a set of TP, FP, and missed detections. The criteria for definition of TP, FP, and working of the proposed detection algorithm is discussed in the next sections.

#### 3.2.1. True Positives (TP)

The algorithm considers every detection as a TP if it is continuously detected by the detector within the temporal window and its average IoU in all the frames in the temporal window is more than or equal to the threshold value λ. Therefore, if the detector marks any FP detection as TP and the detection continues to occur in all the consecutive frames, then our algorithm considers it as a TP detection.

#### 3.2.2. False Positives (FP)

The FP detections are those detections which are not detected in the consecutive frames and their combined IoU is less than the threshold value λ. These FP detections are also declared as FP in the ground truth dataset. The detectors detect them as TP because of camera angle (45°) and the position and angle of the burrow.

#### 3.2.3. Missed Detections

The missed detections are those detections which are TP and are detected in some frames by the detector but missed in some intermediate frames due to position or visibility of the burrow. The missed detections are very important to identify because without identifying them we cannot track a burrow. We can increase the performance of models by recovering the missed detections.

### 3.3. Working of Detection Refinement Algorithm

**Figure 7:**
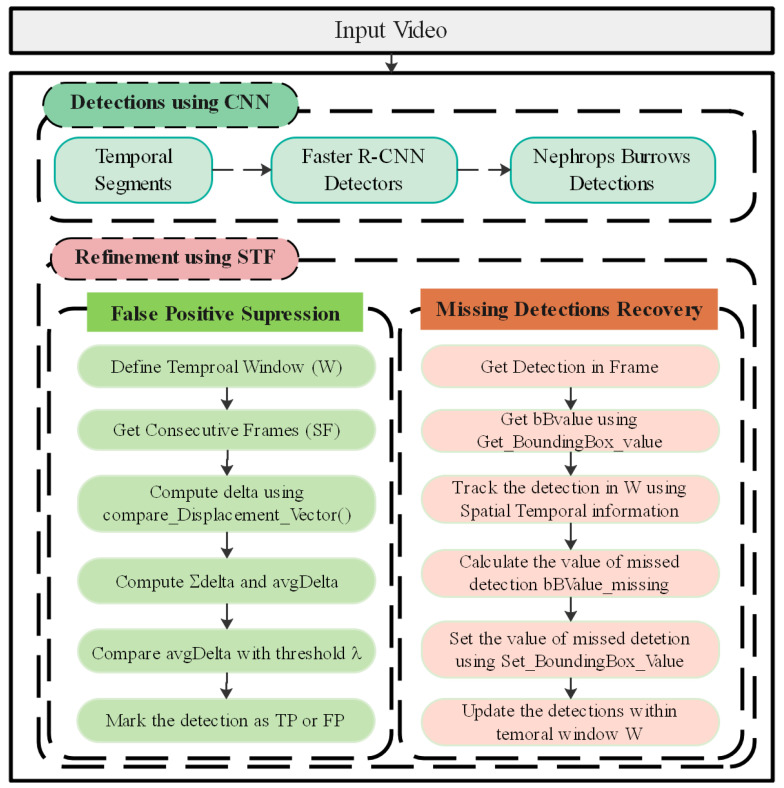
Detection refinement algorithm.

The proposed algorithm is presented in Appendix A and shows the refinement mechanism using the spatial temporal analysis of data. This algorithm is divided into two sections, i.e., suppression of false positives and identification of missed detections. Figure 7 shows the basic processing steps of false positive suppression and missed detection identification and recovery.

#### 3.3.1. Suppression of False Positives

The first step towards the refinement of detections is to suppress the FP. Let *F_i_* = {*B*_1_, *B*_2_,…, *B*_n_} be the frame *i* with *n* detections obtained with a deep learning model. Let *sF* be the set of consecutive frames within a temporal window with size W. The algorithm takes *B_j_* for frame *F_i_* as an input for refinement and provides a refined output as *F_R_*. To suppress the FP in the current frame *i*, we compute the overlapping of each detection *B_j_* of the current frame and the detection in the next frame from *sF.*

The algorithm receives three inputs: an input video with detections *V*, threshold value λ, and temporal window size W. For each detection in the current frame *b* ∈ *B_j_* at frame *F_i_*, we first identify the current detection location in the next frame of *sF* and then compute *δ_κ_* = ΙoU value of current detection with consecutive *k* frame’s detection in *sF* using *Compare_Displacement_Vector(*f*_b_Index_,* fc*_b_Index_)* method (*k =* 1,…, W). Then, *δ*_avg_ = 1/W ∑*δ*_k_ is the estimated average within the temporal window. We marked the detection as FP if *δ*_avg_ < λ, and as TP if otherwise, suppressing the FP. We process the whole video *V* detections in the same way.

#### 3.3.2. Identification of Missed Detections

After refining the detections by suppressing the FP in the previous step, the next step is to identify the missed detections that were missed by our detector. For this purpose, we track each detection *B_j_* ∈ *F_i_* to identify the missed detection. If the detection is found in frame *i +* 1, we continue to track it till the temporal window size W. If the current detection is not tracked in any frame, we mark that as missed detection and store it in the set *indexSet.* To calculate the value of the missed detection, we define the *Set_BoundingBox_Value( )* method. We first compute the location of the missed detection from the *indexSet*. Letting *B_j_* be the current detection and *indexSet_j_* the missed detection, we calculate the accumulative value of detection from the current frame till the *indexSet* location and then calculate the average, called *bBValue_missing*. As we are maintaining the number of frames *N* between the current detection and the missed detection, we calculate the missed detection value by adding the *N* value to the *bBValue_missing.* The missed detections information is then filled and updates the refined output *F_R_*.

## 4. Experiments and Results

In this section, we evaluate the results of different experiments performed using the proposed detection refinement algorithm. We use three different models (Inception, ResNet50, and ResNet101) for training with Gulf of Cadiz dataset. Each model is trained up to 100k iterations, and a log is maintained for each 10k iteration for evaluation.

### 4.1. Quantitative Analysis

In the quantitative analysis, an annotated video with frame rate of 25 fps is used for testing the Inception, ResNet50, and ResNet101 models. The video is divided into five temporal segments, each of one minute. Each temporal segment has 1500 frames.

We record number of detection from each temporal segment by all three models. The detection is then processed through the proposed detection refinement algorithm to identify the TP, FP, and missed detections. Table A2, Table A3, Table A4, Table A5 and Table A6 in Appendix B clearly show the obtained results in each temporal segment by each model and their corresponding improvement by the proposed detection refinement algorithm. The algorithm is run with W = 8, 12, and 16. In each temporal window, the algorithm is tested with λ = 0.3 and 0.4 and finds out the number of TP, FP, missed detection, and F1-score (geometric mean of precision and recall metrics) in each minute of the video.

Table 3 shows the accumulative ground truth (GT), TP, FP, and missed (Miss) detections along with the mean values of precision, recall, and F1-score of each temporal segment. The %Before is the result obtained before applying the STF, while the %After shows the results obtained after applying the refinement algorithm. Table 3 shows that ResNet101 gives the best F1-score in each one of the five temporal segments, followed by ResNet50 and Inception. It was found that a small IoU value of 0.3 is clearly better than 0.4 in terms of precision, recall, and F1-score values because area surrounding burrows is sometimes not well defined for all three models. The effect of window size W shows a trend of better results for smaller values (mostly, W = 8 is better than W = 12 and W = 16).

We performed experiments to find out the accuracy using mean average precision (mAP) after applying the detection refinement algorithm. We selected two different image sets from the third (image set 1) and fifth (image set 2) temporal segments. Each set consists of almost 200 images. Table 4 shows the definition of experiments performed.

**Figure 8:**
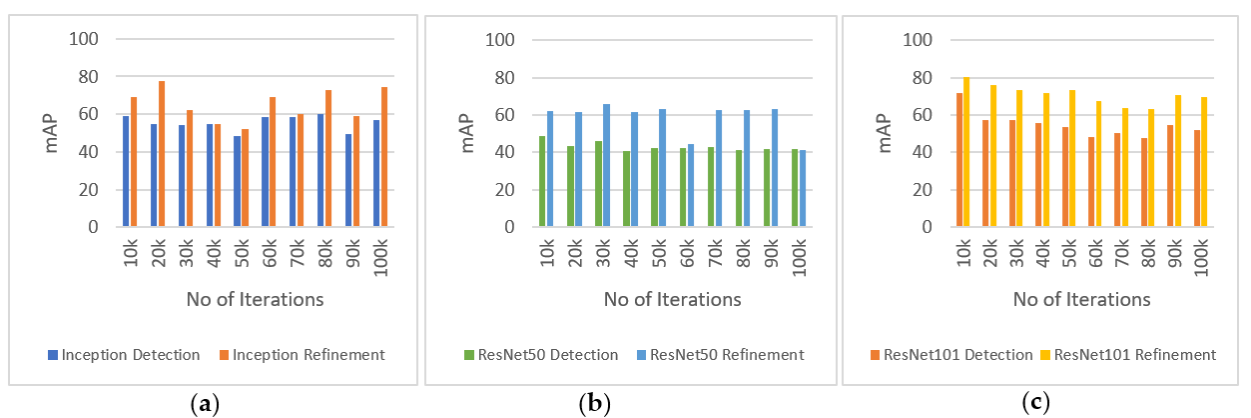
Experiment performed with image set 1 show mean average precision (mAP) of detection refinement with (**a**) detections with Inception model and refinements; (**b**) detections with ResNet50 model and refinements; (**c**) detections with ResNet101 model and refinements.

**Figure 9:**
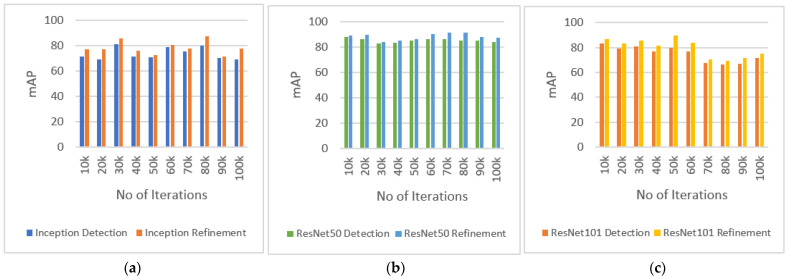
Experiment performed with image set 2 show mean average precision (mAP) of detection refinement with (**a**) detections with Inception model and refinements; (**b**) detections with ResNet50 model and refinements; (**c**) detections with ResNet101 model and refinements.

Figure 8 and Figure 9 show the results of experiments performed on image sets 1 and 2, respectively. The graphs show the results of detections with and without applying the detection refinement algorithm. The performance is evaluated after every 10k iterations. Results clearly show that the mAP increases after applying the refinement algorithm for all three models (Inception (a), ResNet50 (b), and ResNet101 (c)) and iteration number. Figure 8 shows a higher improvement in mAP after applying the proposed refinement algorithm as compared to Figure 9, where some improvement is also achieved, in part due to that image set 1 had obtained a lower mAP before the refinement. Image set 2 has better quality as compared to the images in image set 1, in terms of better appearance of burrows and less camera movement artifacts. This suggest that mAP is quite sensitive to video quality and that the proposed refinement algorithm compensates for this to some degree.

### 4.2. Qualitative Analysis

In this section, we qualitatively analyze the performance of the proposed detection refinement algorithm by applying it to the results obtained from Inception, ResNet50, and ResNet101 models. The red bounding boxes on the images shown in this section are the original detections obtained from the models; green bounding boxes are the recovered missed detections after applying the refinement algorithm, and ground truth data are marked with blue bounding boxes.

**Figure 10:**
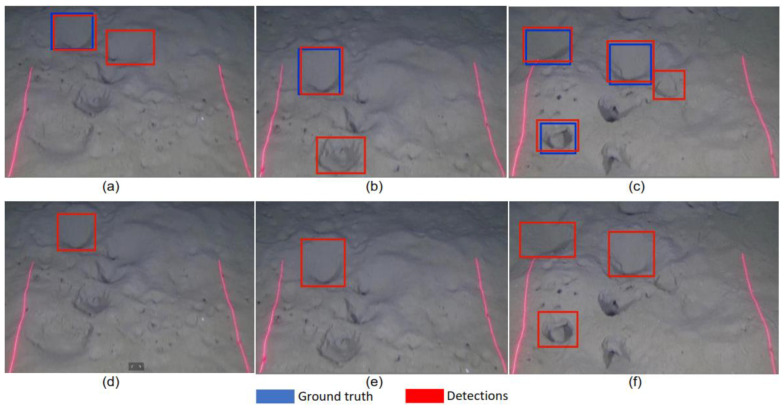
False positive suppression using detection refinement algorithm (**a**–**c**) are the ground truth (blue color bounding boxes), and original detections from Inception model (red color bounding boxes) (**d**–**f**) are the refined detections.

Figure 10 shows a typical example of suppression of FP from the detections obtained from the Inception model. Figure 10a–c shows three frames where all burrows’ entrances are detected correctly but some FP detections are also obtained, yet are suppressed by our proposed algorithm, resulting in a correct detection, which is shown in Figure 10d–f.

**Figure 11:**
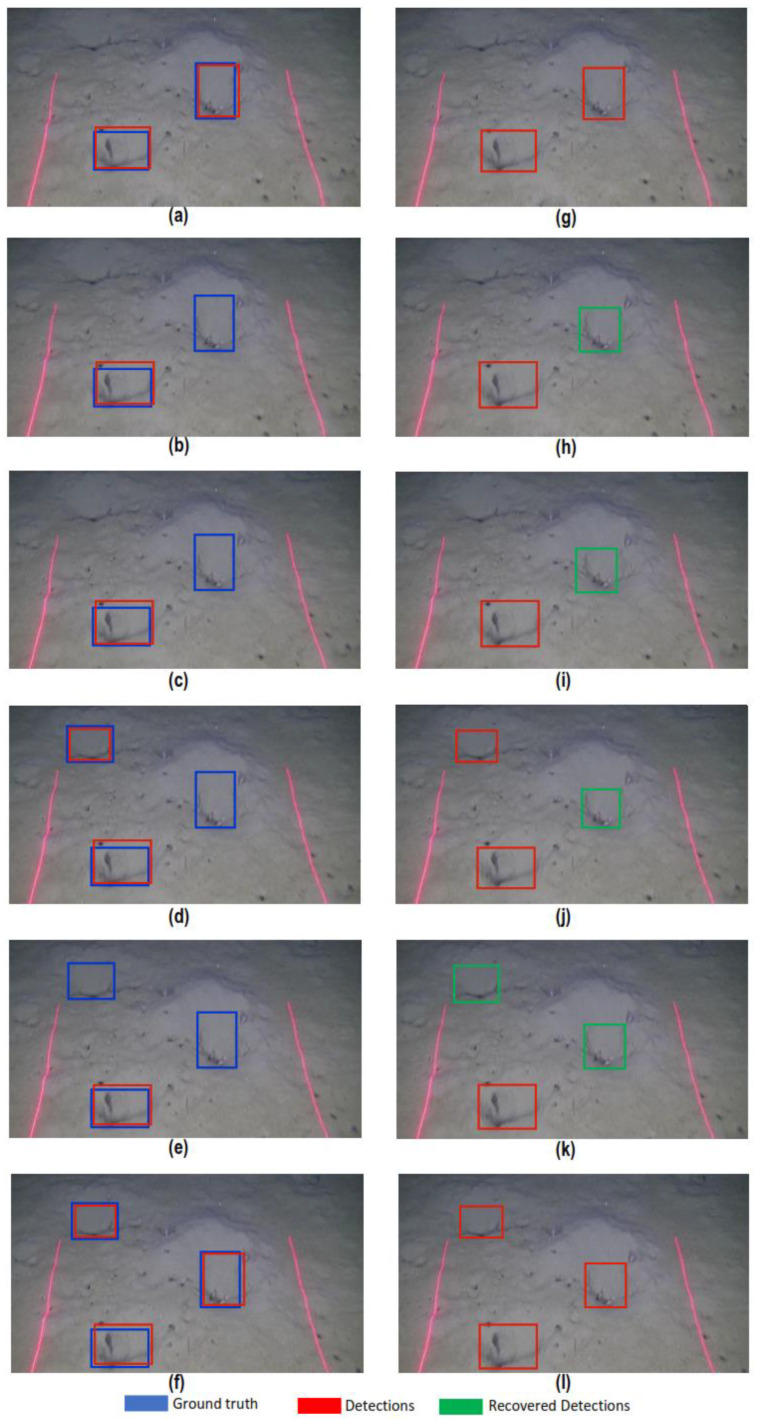
Identification of true positive missed detections. Panels (**a**–**f**) are the original detections from the Inception model, and (**g**–**l**) are the identification of missed detections in the consecutive frames.

A second rectification performed by the proposed detection refinement algorithm is the identification of missed detections. Figure 11 shows an example of six consecutive frames, before (a–f) and after (g–l) the application of this algorithm. Figure 11a shows two *Nephrops* burrows detections but missed one detection in (b–e) which is correctly rectified by the algorithm, as it is shown in the corresponding images (h–k). It can be shown also that ground truth annotations contain a third object in Figure 10d,f, which are correctly detected by the models, but are not shown in Figure 10a–c,e, possibly due to the viewing angle of some frames. However, the identification of missed detections has a good impact on the improvement of accuracy and precision of the results. A similar approach is followed to rectify the detections from ResNet50and ResNet101 models.

## 5. Conclusions

Deep learning algorithms were performed very well on the Gulf of Cadiz dataset in identifying the burrows of *Nephrops norvegicus*. We applied the Faster RCNN algorithms Inception, ResNet50, and ResNet101 for detections. To increase the results accuracy, a spatial–temporal-based detection refinement algorithm was proposed and tested. The proposed algorithm suppresses the false positive detections and recovers the missed true positive detections. The proposed method when integrated with any detector always increased the performance. The performance was calculated using mAP. This mechanism helps marine science experts in the assessment of the abundance of this species.

In future work, we plan to use diverse datasets from UWTV surveys conducted in other *Nephrops* stocks by other countries. We will train the YOLO detectors with more and diverse datasets. In addition, we plan to track the burrows to estimate the abundance of *Nephrops*. We also plan to correlate the spatial and morphological distribution of burrow holes to estimate the number of burrow systems that are present and compare with human inter-observer variability studies.

## Figures and Tables

**Table 1:** Equipment details used in data collection.

Image System
Life Camera
Full HD (1920 × 1080) @ 30 fps Mounting angle 45°
Recording Camera: SONY FDRAX33
4K Ultra HD (3840 × 2160) and Full HD (1920 × 1080) @ 50 fps Mounting angle 45°
Photo camera: SONY ILCE QX1
20.1 MPixel Mounting Angle variable
**Lighting System**
28,640 lumens, distributed in 4 spotlights with individual intensity system TST-OFL 7000 (Thalassatech—Oil Filled LED)
**Photogrammetry System**
3-point lasers (5 mW & λ = 670 nm) forming a triangle of side 70 mm 2-line lasers (200 mW & λ = 670 nm) separated 75 cm (Field of view)
**Auxiliary System**
Battery (Li-ion, size 18,650, 3.7 V & 2400 mAh = capacity 480 Wh)
**Sensors**
Altimeter: Tritech PA500 CTD (conductivity, temperature, and depth): AML Oceanographic MINOS X

**Table 2:** Dataset distribution.

Dataset Distribution			
Functional Unit	Training Images	Testing Images	Total
Gulf of Cadiz Dataset	200 (80%)	48 (20%)	248

**Table 3:** Detections of all temporal segments with refinements. Detections are refined using W = 8, 12, and 16 with λ = 0.3 and 0.4. The refined detection shows total number of TP, FP, and missed detections and F1-score.

	GT = 2359					Recall		Precision		F1-Score	
	W	λ	TP	FP	Miss	%Age Before	%Age After	%Age Before	%Age After	%Age Before	%Age After
Inception	8	0.3	1380	115	256	58.5	69.4	92.3	93.4	71.6	79.6
	8	0.4	1150	345	204	48.7	57.4	76.9	79.7	59.7	66.7
	12	0.3	1316	179	277	55.8	67.5	88.0	89.9	68.3	77.1
	12	0.4	899	596	170	38.1	45.3	60.1	64.2	46.7	53.1
	16	0.3	1308	187	374	55.4	71.3	87.5	90.0	67.9	79.6
	16	0.4	804	691	209	34.1	42.9	53.8	59.4	41.7	49.9
ResNet50	8	0.3	1619	163	356	68.6	90.6	90.9	92.9	78.2	91.8
	8	0.4	1389	393	274	58.9	87.2	77.9	84.0	67.1	85.5
	12	0.3	1557	225	400	66.0	92.5	87.4	90.7	75.2	91.6
	12	0.4	1069	713	239	45.3	85.7	60.0	73.9	51.6	79.4
	16	0.3	1495	287	506	63.4	97.0	83.9	88.9	72.2	92.7
	16	0.4	962	820	260	40.8	86.6	54.0	71.3	46.5	78.2
ResNet101	8	0.3	1894	180	336	80.3	94.5	91.3	92.5	85.5	93.5
	8	0.4	1720	454	262	72.9	84.0	79.1	81.4	75.9	82.7
	12	0.3	1874	265	340	79.4	93.9	87.6	89.3	83.3	91.5
	12	0.4	1267	907	209	53.7	62.6	58.3	61.9	55.9	62.3
	16	0.3	1754	296	421	74.4	92.2	85.6	88.0	79.6	90.1
	16	0.4	1154	1020	228	48.9	58.6	53.1	57.5	50.9	58.1

**Table 4:** Experiments definition for detection refinement.

Experiment	Model	Testing Set
Experiment 1	Inception	Image set 1
Experiment 2	ResNet50	Image set 1
Experiment 3	ResNet101	Image set 1
Experiment 4	Inception	Image set 2
Experiment 5	ResNet50	Image set 2
Experiment 6	ResNet101	Image set 2

## Data Availability

Not applicable.

## Appendix A

**Algorithm A1**: Detection Refinement
**Input Data** *V,* λ*, W*, where *V* is an input video, λ is a threshold value for displacement vector, W is a size of temporal window
**Results** *F_R_* = {*F_1_, F_2_,…, F_n_*}, where *F_R_* is a list of refined frames
**begin**
*F* = Extract_Frames_With_BoundingBox(*V*) *// F = {F_1_, F_2_,…, F_n_}* where *F* is the list of frames and each one *F_i_ = {B_1_, B_2_,…, B_n_}* has *n* bounding boxes *B_j_* = {*x_j_, y_j_, w_j_, h_j_*}, where (*x_j_, y_j_*) are coordinates of initial pixel of the bounding box *j* and *w_j_, h_j_* are width and height.
*T =* Extract_Duration(V) *// T = {T_1_, T_2_, …, T_n_} where T is total time of the video*
**Foreach** *frame f* *∈* F **do**
F_R_ **=** Add_Frame(*f*)
sF **=** Create_Subset_Frame_W_Range(F) // sf is list of frames that need to
compare with current frame till ‘W’ temporal window size
deleteFlag = Set (FALSE)
**Foreach** *boundingbox b* *∈* f **do**
*b_Index* = Get_Bounding_Box_Index(b)
**Foreach** *frame fc* *∈ sF* **do** //*where fc = f+1*
delta += Compare_Displacement_Vector (f*_b_Index_*, fc*_b_Index_*)
**endFor**
avgDelta = delta/W
**if** avgDelta < λ **then**
deleteFlag = Set (TRUE)
**endif**
**if** deleteFlag is FALSE **then**
F_R_**=** Add_ Bounding_Box_in_Frame (*f,* f*_b_Index_)*
**endif**
**endFor**
**Foreach** *boundingbox b* *∈* f **do**
indexSet **=** Identify_Missing_Detection(b, F_R_*)*
**endFor**
lastIndex = 0
**for** index in F_R_ do
**if** index is in indexSet
j = index
for lastIndex to j
bBValue += Get_BoundingBox_Value(b, f _lastIndex_*)*
*endfor*
*bBValue_missing =* bBValue/j
Set_BoundingBox_Value(b, fj, *bBValue_missing)*
lastIndex = j;
**endif**
**endFor**
**endFor**
**return** F_R_
**end**

## Appendix B

**Table A1:** Underwater object detection with key findings.

Author	Year	Approach	Object Detection	Dataset	Performance Parameters
Li et al.	2015	Deep Convolutional Network	Marine Objects	ImageCLEF_Fish_TS dataset 24272 Images	mAP
Villon et al.	2016	HOG, SVM and Deep Learning	Fish Detection	Fish4Knowledge 13000 fish thumbnails	Precision, Recall, F-Score
Rathi et al.	2018	Faster R-CNN (ZF Net, CNN-M, VGG16)	Fishes & crustacean species	Fish4Knowledge 27,142 Images	AP
Xu et al.	2018	YOLO	Fishes	3 datasets	mAP
Mandal et al.	2018	Faster R-CNN	Fishes	Uni of Sunshine Coast 12365 Images	mAP
Jalal et al.	2020	YOLO based Hybrid approach	Fish Classification	LifeCLEF 2015 93 Videos	F-Score
Sung et al.	2017	YOLO	Fish detection	892 Images	Precision, Recall, FPS
Jager et al.	2016	CNN AlexNet	Fish Classification	LifeCLEF2015	AP, Precision, Recall,
Zhuang et al.	2017	ResNet-10	Underwater Species	SEACLEF2017	AP
Zhao et al.	2021	Composed FishNet	Fish and Underwater Species detections	SeaCLEF 2017 200,000 images	AP, F-Measure
Labao et al.	2019	Multilevel R-CNN	Fish detection	300 Underwater Images	Precision, Recall, F-Score
Salman et al.	2019	Two stage R-CNN	Fish detection	Fish4Knowledge, LCF-15	Precision, Recall, F-Score
Lee et al.	2016	Three layers CNN	Plankton detection	WHOI-Plankton database 3.2 million Images	F1-Score

**Table A2:** Detections and refinement results of 1st temporal segment.

1st Temporal Segment											
	GT = 255					Recall		Precision		F1-Score	
	W	λ	TP	FP	Miss	%Age Before	%Age After	%Age Before	%Age After	%Age Before	%Age After
Inception	8	0.3	166	9	13	65.1	70.2	94.9	95.2	77.2	80.8
	8	0.4	149	26	12	58.4	63.1	85.1	86.1	69.3	72.9
	12	0.3	165	10	15	64.7	70.6	94.3	94.7	76.7	80.9
	12	0.4	68	107	9	26.7	30.2	38.9	41.8	31.6	35.1
	16	0.3	163	12	41	63.9	80.0	93.1	94.4	75.8	86.6
	16	0.4	66	109	19	25.9	33.3	37.7	43.8	30.7	37.9
ResNet50	8	0.3	188	20	31	73.7	85.9	90.4	91.6	81.2	88.7
	8	0.4	177	31	20	69.4	77.3	85.1	86.4	76.5	81.6
	12	0.3	186	22	43	72.9	89.8	89.4	91.2	80.3	90.5
	12	0.4	110	98	19	43.1	50.6	52.9	56.8	47.5	53.5
	16	0.3	175	33	41	68.6	84.7	84.1	86.7	75.6	85.7
	16	0.4	93	115	12	36.5	41.2	44.7	47.7	40.2	44.2
ResNet101	8	0.3	217	26	24	85.1	94.5	89.3	90.3	87.1	92.3
	8	0.4	164	79	20	64.3	72.2	67.5	70.0	65.9	71.0
	12	0.3	188	55	28	73.7	84.7	77.4	79.7	75.5	82.1
	12	0.4	100	143	18	39.2	46.3	41.2	45.2	40.2	45.7
	16	0.3	181	62	21	71.0	79.2	74.5	76.5	72.7	77.8
	16	0.4	96	147	13	37.6	42.7	39.5	42.6	38.6	42.7

**Table A3:** Detections and refinement results of 2nd temporal segment.

2nd Temporal Segment											
	GT = 585					Recall		Precision		F1-Score	
	W	λ	TP	FP	Miss	%Age Before	%Age After	%Age Before	%Age After	%Age Before	%Age After
Inception	8	0.3	398	33	61	68.0	78.5	92.3	93.3	78.3	85.2
	8	0.4	324	107	46	55.4	63.2	75.2	77.6	63.8	69.7
	12	0.3	393	38	73	67.2	79.7	91.2	92.5	77.4	85.6
	12	0.4	271	160	41	46.3	53.3	62.9	66.1	53.3	59.0
	16	0.3	393	38	115	67.2	86.8	91.2	93.0	77.4	89.8
	16	0.4	269	162	68	46.0	57.6	62.4	67.5	53.0	62.2
ResNet50	8	0.3	420	45	105	71.8	89.7	90.3	92.1	80.0	90.9
	8	0.4	306	159	85	52.3	66.8	65.8	71.1	58.3	68.9
	12	0.3	404	61	114	69.1	88.5	86.9	89.5	77.0	89.0
	12	0.4	241	224	78	41.2	54.5	51.8	58.7	45.9	56.6
	16	0.3	363	102	168	62.1	90.8	78.1	83.9	69.1	87.2
	16	0.4	232	233	104	39.7	57.4	49.9	59.1	44.2	58.2
ResNet101	8	0.3	441	31	103	75.4	93.0	93.4	94.6	83.4	93.8
	8	0.4	433	139	89	74.0	89.2	75.7	79.0	74.8	83.8
	12	0.3	468	49	103	80.0	97.6	90.5	92.1	84.9	94.8
	12	0.4	309	263	68	52.8	64.4	54.0	58.9	53.4	61.6
	16	0.3	415	57	145	70.9	95.7	87.9	90.8	78.5	93.2
	16	0.4	300	272	89	51.3	66.5	52.4	58.9	51.9	62.4

**Table A4:** Detections and refinement results of 3rd temporal segment.

3rd Temporal Segment											
	GT = 480					Recall		Precision		F1-Score	
	W	λ	TP	FP	Miss	%Age Before	%Age After	%Age Before	%Age After	%Age Before	%Age After
Inception	8	0.3	163	23	45	34.0	43.3	87.6	90.0	48.9	58.5
	8	0.4	132	54	37	27.5	35.2	71.0	75.8	39.6	48.1
	12	0.3	160	26	47	33.3	43.1	86.0	88.8	48.0	58.1
	12	0.4	106	80	30	22.1	28.3	57.0	63.0	31.8	39.1
	16	0.3	159	27	46	33.1	42.7	85.5	88.4	47.7	57.6
	16	0.4	64	122	28	13.3	19.2	34.4	43.0	19.2	26.5
ResNet50	8	0.3	291	43	87	60.6	78.8	87.1	89.8	71.5	83.9
	8	0.4	269	65	69	56.0	70.4	80.5	83.9	66.1	76.6
	12	0.3	280	54	106	58.3	80.4	83.8	87.7	68.8	83.9
	12	0.4	203	131	59	42.3	54.6	60.8	66.7	49.9	60.0
	16	0.3	274	60	114	57.1	80.8	82.0	86.6	67.3	83.6
	16	0.4	181	153	55	37.7	49.2	54.2	60.7	44.5	54.3
ResNet101	8	0.3	354	40	105	73.8	95.6	89.8	92.0	81.0	93.8
	8	0.4	335	59	88	69.8	88.1	85.0	87.8	76.7	87.9
	12	0.3	368	46	111	76.7	99.8	88.9	91.2	82.3	95.3
	12	0.4	302	92	64	62.9	76.3	76.6	79.9	69.1	78.0
	16	0.3	325	45	136	67.7	96.0	87.8	91.1	76.5	93.5
	16	0.4	268	126	79	55.8	72.3	68.0	73.4	61.3	72.8

**Table A5:** Detections and refinement results of 4th temporal segment.

4th Temporal Segment											
	GT = 468					Recall		Precision		F1-Score	
	W	λ	TP	FP	Miss	%Age Before	%Age After	%Age Before	%Age After	%Age Before	%Age After
Inception	8	0.3	304	24	64	65.0	78.6	92.7	93.9	76.4	85.6
	8	0.4	280	48	51	59.8	70.7	85.4	87.3	70.4	78.2
	12	0.3	296	32	67	63.2	77.6	90.2	91.9	74.4	84.1
	12	0.4	235	93	48	50.2	60.5	71.6	75.3	59.0	67.1
	16	0.3	293	35	72	62.6	78.0	89.3	91.3	73.6	84.1
	16	0.4	206	122	43	44.0	53.2	62.8	67.1	51.8	59.4
ResNet50	8	0.3	330	28	66	70.5	84.6	92.2	93.4	79.9	88.8
	8	0.4	284	74	50	60.7	71.4	79.3	81.9	68.8	76.3
	12	0.3	327	31	81	69.9	87.2	91.3	92.9	79.2	90.0
	12	0.4	247	111	50	52.8	63.5	69.0	72.8	59.8	67.8
	16	0.3	325	33	98	69.4	90.4	90.8	92.8	78.7	91.6
	16	0.4	232	126	49	49.6	60.0	64.8	69.0	56.2	64.2
ResNet101	8	0.3	388	42	50	82.9	93.6	90.2	91.3	86.4	92.4
	8	0.4	352	78	37	75.2	83.1	81.9	83.3	78.4	83.2
	12	0.3	387	43	57	82.7	94.9	90.0	91.2	86.2	93.0
	12	0.4	247	183	38	52.8	60.9	57.4	60.9	55.0	60.9
	16	0.3	380	50	61	81.2	94.2	88.4	89.8	84.6	92.0
	16	0.4	232	198	31	49.6	56.2	54.0	57.0	51.7	56.6

**Table A6:** Detections and refinement results of 5th temporal segment.

5th Temporal Segment											
	GT = 571					Recall		Precision		F1-Score	
	W	λ	TP	FP	Miss	%Age Before	%Age After	%Age Before	%Age After	%Age Before	%Age After
Inception	8	0.3	349	26	73	61.1	73.9	93.1	94.2	73.8	82.8
	8	0.4	265	110	58	46.4	56.6	70.7	74.6	56.0	64.3
	12	0.3	302	73	75	52.9	66.0	80.5	83.8	63.8	73.8
	12	0.4	219	156	42	38.4	45.7	58.4	62.6	46.3	52.8
	16	0.3	300	75	100	52.5	70.1	80.0	84.2	63.4	76.5
	16	0.4	199	176	51	34.9	43.8	53.1	58.7	42.1	50.2
ResNet50	8	0.3	390	27	67	68.3	80.0	93.5	94.4	78.9	86.6
	8	0.4	353	64	50	61.8	70.6	84.7	86.3	71.5	77.6
	12	0.3	360	57	56	63.0	72.9	86.3	87.9	72.9	79.7
	12	0.4	268	149	33	46.9	52.7	64.3	66.9	54.3	59.0
	16	0.3	358	59	85	62.7	77.6	85.9	88.2	72.5	82.6
	16	0.4	224	193	40	39.2	46.2	53.7	57.8	45.3	51.4
ResNet101	8	0.3	494	41	54	86.5	96.0	92.3	93.0	89.3	94.5
	8	0.4	436	99	28	76.4	81.3	81.5	82.4	78.8	81.8
	12	0.3	463	72	41	81.1	88.3	86.5	87.5	83.7	87.9
	12	0.4	309	226	21	54.1	57.8	57.8	59.4	55.9	58.6
	16	0.3	453	82	58	79.3	89.5	84.7	86.2	81.9	87.8
	16	0.4	258	277	16	45.2	48.0	48.2	49.7	46.7	48.8
